# Porous Activated Carbon from Lignocellulosic Agricultural Waste for the Removal of Acetampirid Pesticide from Aqueous Solutions

**DOI:** 10.3390/molecules25102339

**Published:** 2020-05-17

**Authors:** Somaia G. Mohammad, Sahar M. Ahmed, Abd El-Galil E. Amr, Ayman H. Kamel

**Affiliations:** 1 Agriculture Research Center (ARC), Central Agricultural Pesticides Laboratory, Pesticide Residues and Environmental Pollution Department, Dokki, Giza 12618, Egypt; sommohammad2015@gmail.com; 2 Petrochemical Department, Egyptian Petroleum Research Institute, Ahmed El-Zomor St., Nasr City, Cairo 11727, Egypt; saharahmed92@hotmail.com; 3 Pharmaceutical Chemistry Department, Drug Exploration & Development Chair (DEDC), College of Pharmacy, King Saud University, Riyadh 11451, Saudi Arabia; 4 Applied Organic Chemistry Department, National Research Center, Dokki, Giza 12622, Egypt; 5 Chemistry Department, Faculty of Science, Ain Shams University, Abbasia, Cairo 11566, Egypt

**Keywords:** green-removal, tangerine peels activated carbon, agriculture waste, acetamiprid pesticide

## Abstract

A facile eco-friendly approach for acetampirid pesticide removal is presented. The method is based on the use of micro- and mesoporous activated carbon (TPAC) as a natural adsorbent. TPAC was synthesized via chemical treatment of tangerine peels with phosphoric acid. The prepared activated carbon was characterized before and after the adsorption process using Fourier- transform infrared (FTIR), X-ray diffraction (XRD), particle size and surface area. The effects of various parameters on the adsorption of acetampirid including adsorbent dose (0.02–0.2 g), pH 2–8, initial adsorbate concentration (10–100 mg/L), contact time (10–300 min) and temperature (25–50 °C) were studied. Batch adsorption features were evaluated using Langmuir and Freundlich isotherms. The adsorption process followed the Langmuir isotherm model with a maximum adsorption capacity of 35.7 mg/g and an equilibration time within 240 min. The adsorption kinetics of acetamiprid was fitted to the pseudo-second-order kinetics model. From the thermodynamics perspective, the adsorption was found to be exothermic and spontaneous in nature. TPAC was successfully regenerated and reused for three consecutive cycles. The results of the presented study show that TPAC may be used as an effective eco-friendly, low cost and highly efficient adsorbent for the removal of acetamiprid pesticides from aqueous solutions.

## 1. Introduction

Pesticides are carcinogens in nature, and because of their toxicity, they pollute both land and water environments. Pesticide detection in groundwater has increased interest in finding suitable techniques used for their removal from aqueous solution to minimize their concentrations to the permissible level [1].

**Figure 1:**
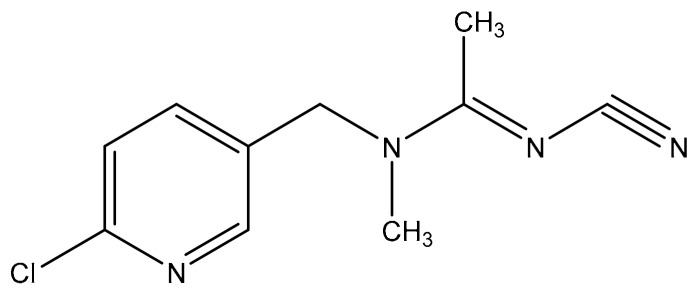
Chemical structure of acetamiprid.

Acetamiprid (Figure 1) belongs to a new neonicotinoid class of systemic broad-spectrum insecticides. Due to the relatively low and chronic mammalian toxicity, and no long-term cumulative toxicity, it has been used to take the place of organophosphorus and other conventional insecticides for controlling the insects such as Hemiptera, Thysanoptera and Lepidoptera in agricultural products [2]. It is also used to protect crops, especially vegetables, fruits from different insect pests, such as aphids and thrips.

Acetamiprid is widely used due to its high water-solubility, low toxicity for humans and high activity against insects [3]. It is susceptible to be found in water according to the United States Environmental Protection Agency (EPA) due to its high solubility [4]. It contaminates various matrices (water, soil, plants and aquatic species) [5]. In addition, it revealed potential health risk to human beings who are exposed to the primary route of food and water polluted by acetamiprid. However, scientific literature regarding the removal of acetamiprid by means of non-conventional treatment technologies is still incomplete [6].

There are many different methods used for the removal of pesticides from aqueous solutions such as oxidation, photocatalysis, electrochemical and adsorption [7]. The biosorption process is a physicochemical and alternative phenomenon and has many benefits such as abundant in nature, cheap, simplicity, efficient and recycling [6,7]. The mechanism of adsorption involved adsorption via ion exchange, chelation and complexation. It depends on different factors such as the chemical nature of the pollutants, type of the biosorbents, temperature, pH and ionic strength [8].

Different methods were reported for acetamiprid removal from aqueous solutions such as advanced oxidation processes (AOPs) Fenton-based treatments [9,10], heterogeneous photocatalysis [11,12], static sorption based on different clays [13] and other related technologies like the innovative low temperature plasma [14] have been published in the last few years. All of these methods demonstrated their potential towards the efficient removal of acetamiprid from different water matrices.

Activated carbon is considered as an effective adsorbent for removing organic contaminants from aqueous media because of its large specific surface area, excellent stability, environment-friendly, favorable physical/chemical surface characteristics, easy preparation, management and high removal efficiency [15,16]. Activated carbon has been considered to be a promising and effective sorbent for removing of toxic metals [17] and pesticides such as oxamyl [18,19], butachlor [20], diazinon [21] and carbofuran [22] from aqueous solution. However, limited works have been found for the removal of acetamiprid insecticide by agricultural waste [23].

The adsorption efficiency of carbon materials can be enhanced through chemical activation using phosphoric acid. The use of this acid can increase the carbon content to (35%–50%) in the adsorbent. In addition, it is easy to be recovered and has less environmental impacts from its use. The role of the phosphoric acid in converting the precursor to the activated carbon is promoting the decomposition of the precursor and forming the cross-linked structure at low temperatures that make pores more open and then enlarging the surface area [17].

In the present study, porous activated carbon from tangerine peel waste was introduced as an effective biosorbent for acetamiprid pesticide removal from aqueous media. Phosphoric acid was used for the chemical activation of these agriculture wastes. The prepared activated carbon was characterized by FTIR, surface area, XRD and particle size. Different parameters were used in this study through batch adsorption such as different mass, pH, initial concentration of acetamiprid pesticide and contact time. The removal capacity was studied by fitting the adsorption data for isotherm, kinetics and thermodynamics.

## 2. Results and Discussion

### 2.1. Characterization of TPAC

#### 2.1.1. FTIR Analysis

**Figure 2:**
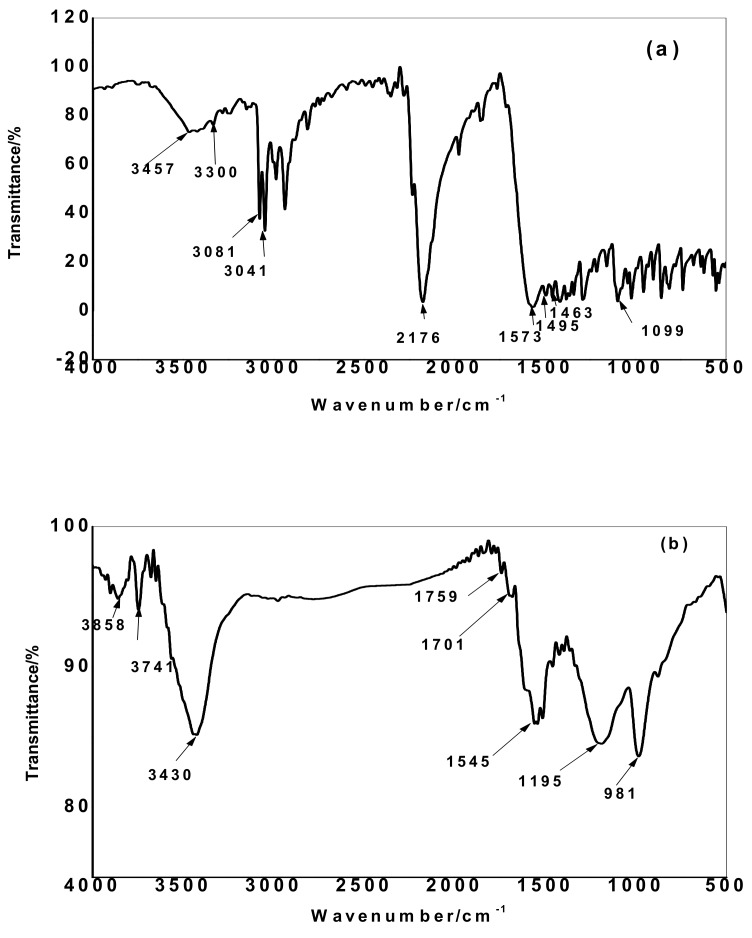

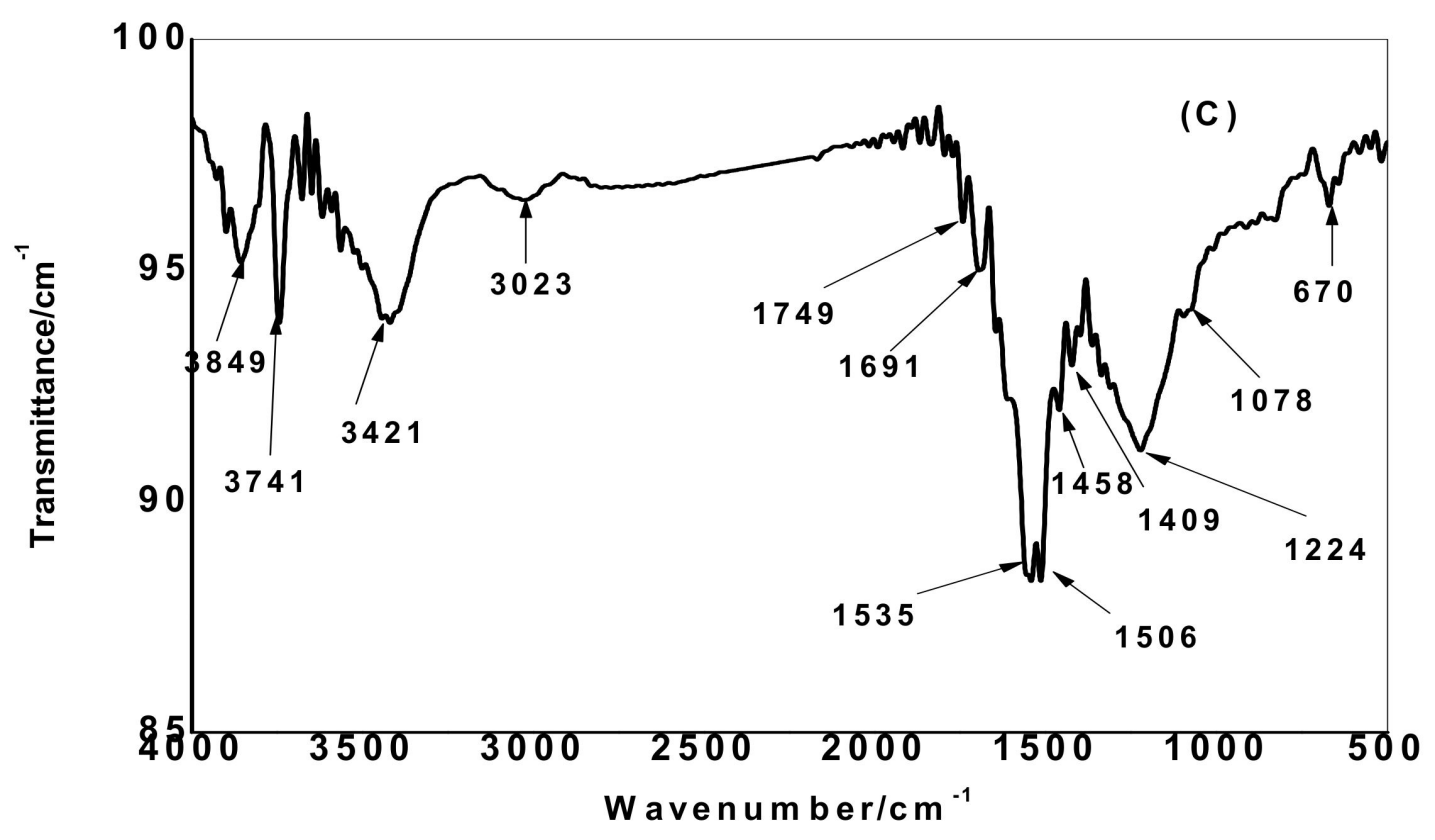
Fourier- transform infrared (FT-IR) of micro and mesoporous activated carbon (TPAC) (**a**) acetamiprid (**b**) before, (**c**) after the adsorption of acetamiprid.

The FT-IR spectrum of acetamiprid is shown in Figure 2a. Stretching vibrations of both –CN and C=N groups were observed at 2175 and 1567 cm^−1^, respectively. Sharp stretching vibrations of C–H bond were observed at 2600–3000 cm^−1^. Olefinic =CH- groups in the pyridine group were observed at 1495 cm^−1^ and 1463 cm^−1^, respectively [24]. Stretching C–Cl bond is located at 1099 cm^−1^. Typical stretching vibrations for N–H and C–N bonds are located at 3300–3487 cm^−1^ and 1341 cm^−1^, respectively. The FTIR spectra of the TPAC before and after adsorption of acetamiprid were shown in Figure 2b,c. The data showed a broad band at 3423 cm^−1^ which is assigned to stretching vibration of O–H bond. The assigned C–H peak of acetamiprid appeared as a broad weak peak at 3028 cm^−1^. The C=N and olefinic =CH- groups in acetamiprid appeared at 1567 and 1495 cm^−1^ are now located in Figure 2c at 1535 and 1506 cm^−1^, respectively. The stretching C–Cl bond which is located at 1099 cm^−1^ is now appeared as weak peak at 1078 cm^−1^. The band at 1691 cm^−1^ is due to the amide group. Stretching vibration of C=O bond in the carboxylic group is observed at 1742 cm^−1^. The difference in spectra of TPAC before and after adsorption of acetamiprid is attributed to the interaction between the hydroxyl, amine and carboxylic groups of TPAC and the acetamiprid pesticide.

#### 2.1.2. Specific Surface Area and Particle Size

**Figure 3:**
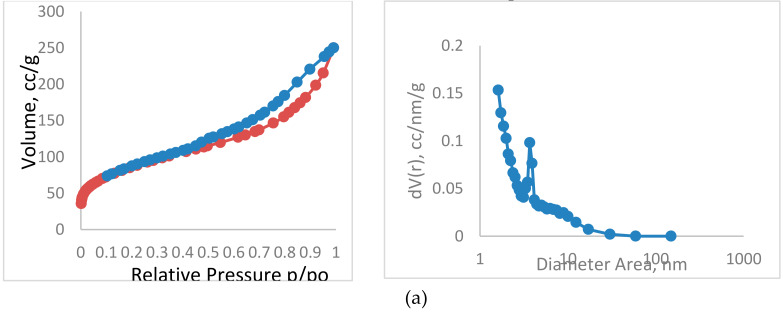

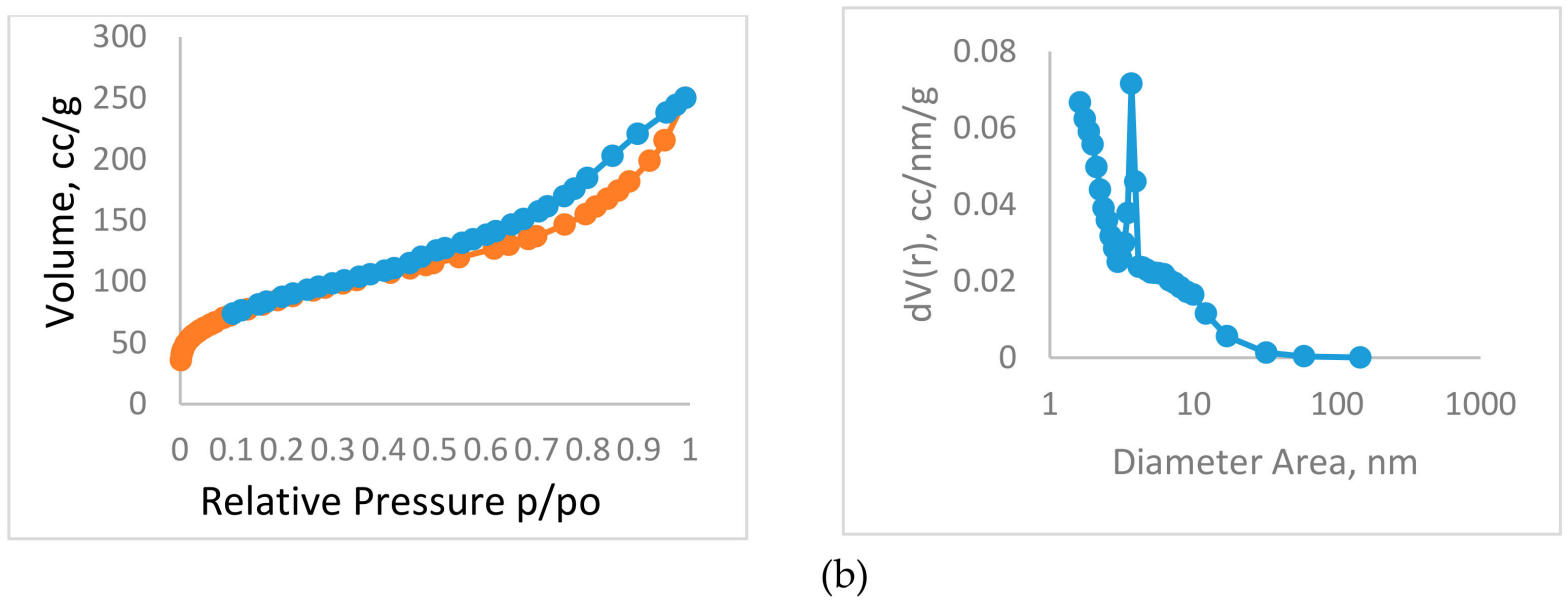
(**a**) Nitrogen adsorption–desorption isotherms and the corresponding pore size distribution curve of the prepared TPAC; (**b**) nitrogen adsorption–desorption isotherms and the corresponding pore size distribution curve of the prepared TPAC after adsorption of acetamiprid.

The N_2_ adsorption/desorption isotherms and pore size distribution curves were evaluated to characterize the behavior of the prepared TPAC before and after adsorption of acetamiprid (Figure 3). The obtained adsorption isotherm of TPAC sample can be deemed as a combination of type I and IV isotherm curves. As shown in Figure 3a, the Type I isotherm is concave to the *P/P*^0^ axis and the amount adsorbed approaches a limiting value. This limiting uptake is governed by the accessible micropore volume rather than by the internal surface area. A steep uptake at very low *P/P*^0^ is due to enhanced adsorbent–adsorptive interactions in narrow micropores (micropores of molecular dimensions), resulting in micropore filling at very low *P/P*^0^. Type IV isotherms are given by mesoporous adsorbents. The adsorption behavior in mesopores is determined by the adsorbent–adsorptive interactions and also by the interactions between the molecules in the condensed state. In this case, the initial monolayer–multilayer adsorption on the mesopore walls [25]. According to *t*-plot method, the textural features of TPAC before and after acetamiprid adsorption were presented in Table 1. From the date obtained, the structure of TPAC was a combination of meso- and micropores with a larger proportion of mesoporous [26]. It is observed that the total surface area and the total pore volume of TPAC after the adsorption of the pesticide decreased from 687.8 to 296.4 m^2^/g and from 0.64 to 0.38 cc/g, respectively. This indicates that the free pores are congested by the pesticide molecules and indicates the existence of both micro- and meso-porous in the TPAC structure.

**Figure 4:**
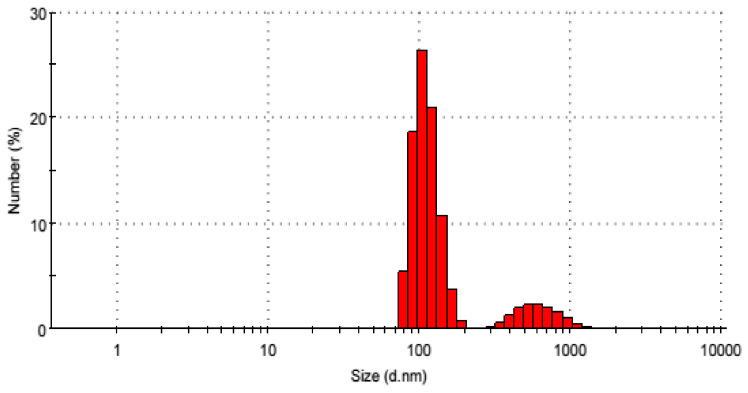
The Particle size of TPAC.

Dynamic light scattering (DLS) is used to determine the particle size distribution of the prepared TPAC as shown in Figure 4. The data showed that about 62.4% of the particles have sizes between 80 and 205 nm with a mean size of 142.5 nm. It is also showed that about 25% of the particles have sizes lower than or equal to 100 nm; 4.2% of the particles have sizes between 295 and 1055 nm, and less than 0.5% have sizes greater than 1000 nm.

#### 2.1.3. XRD Analysis

**Figure 5:**
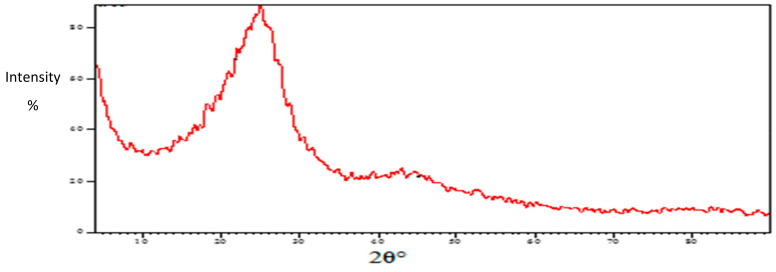
X-ray diffraction (XRD) of TPAC.

The crystal structure and the phase purity of the prepared TPAC were examined by XRD analysis. The XRD pattern for TPAC is shown in Figure 5. It exhibited one broad diffraction peak at 2θ = 25.18 (d002) and a small peak at 45.85 (d100) which are attributed to the presence of graphite crystallite in TPAC. The diffraction patterns agree well with the standard JCPDS for the graphite phase with the hexagonal structure (JCPDS no. 7440-44-0). 

### 2.2. Effect of Different Factors on the Adsorption Process

**Figure 6:**
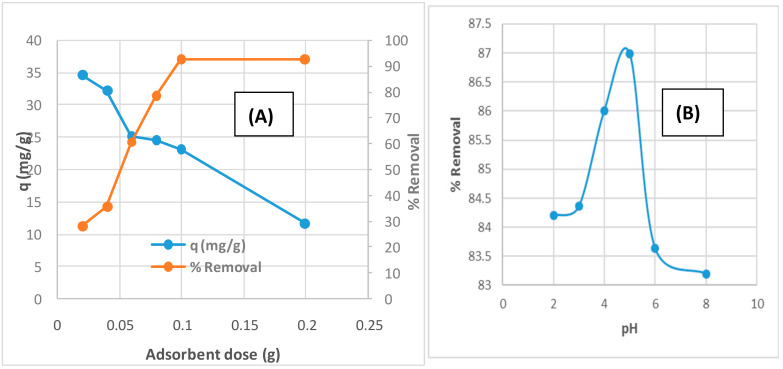

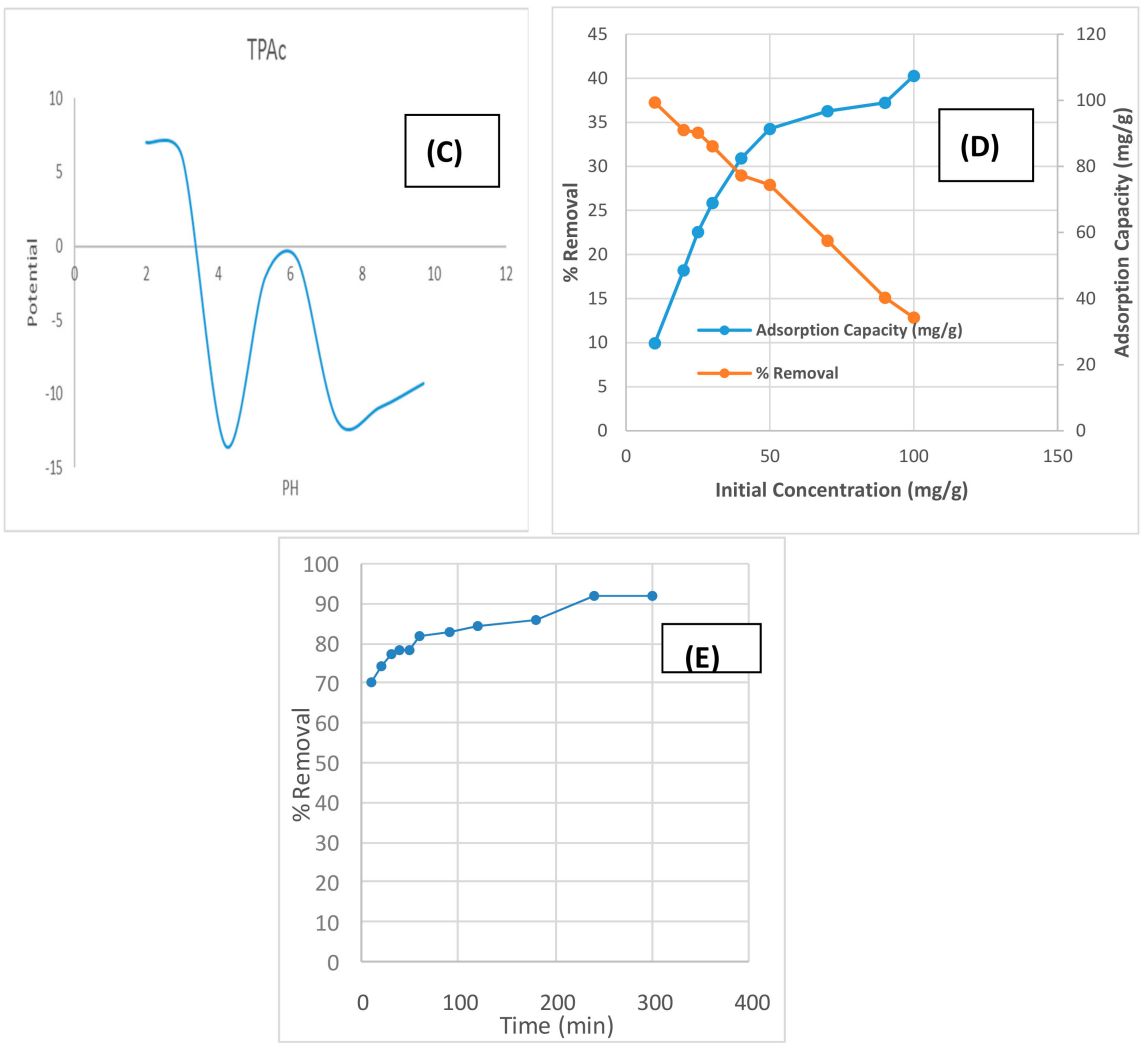
Effect of (**A**) adsorbent dose (initial concentration 25 mg/L, stirring 200 rpm and contact time 5 h); (**B**) pH (initial concentration 25 ppm, mass 0.1 g and contact time 5 h); (**C**) zeta potential of TPAC; (**D**) initial concentration of acetamiprid (mass 0.1 g and contact time 5 h); and (**E**) contact time (mass 0.1 g, stirring 200 rpm and initial concentration 25 ppm) on the removal of acetamiprid by TPAC.

The effect of different parameters on the removal of acetamiprid pesticide by TPAC such as adsorbent dose, pH and initial concentrations of acetamiprid and contact time was investigated. Adsorbent dosage is an important parameter in the adsorption process because it determines the sorbent–sorbate equilibrium of the system and the capacity of the adsorbent for a given concentration [27]. The effect of adsorbent dose on the removal of acetamiprid pesticide by TPAC was studied at different adsorbent doses varying from 0.02–0.2 g/100 mL as shown in Figure 6a. As the adsorbent dose of TPAC increases, the percentage removal of acetamiprid increased from 27.8% to 92.5%. This can be attributed to the increase in the number of active sites [28]. At higher adsorbent dosage, there is incomplete usage of the adsorption sites due to the formation of aggregates. In contrast to the percentage removal, the adsorption capacity of acetamiprid by TPAC was declined in the range of 34.7–11.5 mg/g with increasing adsorbent dose. It was noted that after an adsorbent dosage of 0.1 g, the adsorption efficiency did not show remarkable increase that indicates the saturation of the adsorption sites. An adsorbent dose of 0.1 g seems to be optimum for the removal of acetamiprid experiments by TPAC.

The effect of pH on the removal of acetamiprid pesticide by TPAC was studied at the pH range from 2–8 at initial concentration of 25 µg/mL and adsorbent dose 0.1 g. The effect of pH on the removal of acetamiprid onto TPAC from aqueous solution at different pH values (2–8) and zeta potential of TPAC are shown in Figure 6b,c. The removal percentage of acetamiprid reaches its maximum value (86.99%) at pH 5.0. At pH > 5, the percentage removal decreases and reaches 83.2% at pH 8. These results indicated that pH 5 is suitable for the maximum removal of acetamiprid than the higher pH values. Figure 6c illustrates that PZC for TPAC was found to be 6.0 and its surface carries a negative charge at pH 5. Hence, pH 5 was chosen for all subsequent removal steps of positively charged acetamiprid molecules. The maximum sorption is attributed to the strong van der Waals attraction between the surface groups and pesticide molecules [29].

The effect of the initial concentration of acetamiprid was carried out by increasing acetamiprid concentration from 10–100 µg/mL. As shown in Figure 6D, the adsorption capacity increased from 9.94 to 36.31 mg/g and the removal efficiency decreased from 99.37% to 40.275% upon changing the initial concentration of the pesticide from 10 to 100 mg/L. These results showed that the adsorption of acetamiprid pesticide by TPAC depends on the initial concentration. Similar trends have been reported by the previous studies using activated carbon from pumpkin seed hulls for the removal of 2,4-dichlorophenoxyacetic acid [30,31].

Contact time is an important parameter for the removal of acetamiprid by TPAC. As shown in Figure 6E, the adsorption process of acetamiprid by TPAC was rapid in the first 10 min with percentage removal of 70.1% and reaches its maximum value (91%) after 240 min. The adsorption of acetamiprid attained equilibrium at 240 min due to the fully occupied of the available adsorption sites on the surface of the adsorbent. Further increase in the contact time did not affect the adsorption.

### 2.3. Adsorption Kinetic Modeling

The kinetics of acetamiprid adsorption by TPAC was studied by two common models, pseudo-first-order [32] and pseudo-second-order models [33].

The pseudo-first-order is given by Equation (1).

*Log* (*q_e_* − *q_t_*) = *log q_e_* − (*k_1_*/2.303) *t* (1)

**Figure 7:**
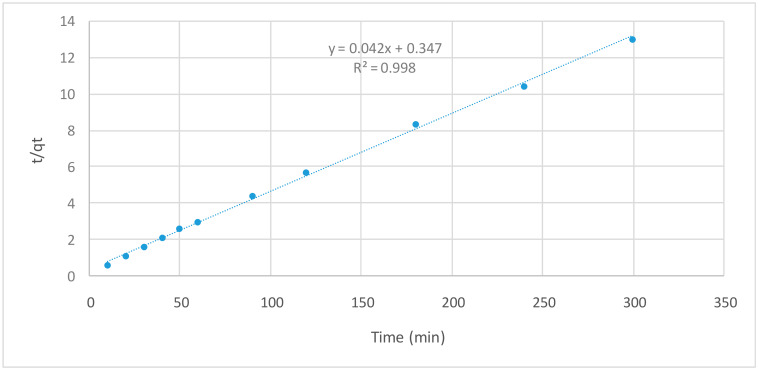
Pseudo-second-order kinetic model for adsorption of acetamiprid by TPAC.

The pseudo-second-order is given by Equation (2):

*t/q_t_* = 1/*k*_2_*q*_*e*2_ + (1/*q_e_*) *t* (2)

where *q_e_* and *q_t_* are the amount of acetamiprid adsorbed by TPAC at equilibrium and at time *t*, respectively. The adsorption rate constants of pseudo-first and pseudo-second-order were represented as *k*_1_ and *k*_2_, respectively. The kinetic parameters are given in Table 2. As can be seen from Figure 7, the pseudo-second-order kinetic model adsorption capacity calculated is 23.364 mg/g. The low value of *R*^2^ for the pseudo-first-order kinetic model could be an indication that the rate of the adsorption of acetamiprid by TPAC depends on both concentration and time [34]. The results showed that (*R*^2^) values of the pseudo-second-order kinetic model were closer to unity, indicating that the adsorption of acetamiprid by TPAC followed the pseudo-second-order kinetic model. Similar trend was observed for the adsorption of bentazon on activated carbon prepared from *Lowosoniainerm* wood [35].

### 2.4. Adsorption Isotherms Modeling

The adsorption isotherms are important to describe the interaction between adsorbate and adsorbent. In this study, different isotherm models were used to study the adsorption of acetamiprid pesticide onto TPAC. The most commonly applied isotherms such as Langmuir [36] and Freundlich [37] isotherm models were tested. The linear form of Langmuir isotherm is given by Equation (3).

1/*Q_t_* = (1/*X_m_ b*) *C_t_* + 1/*X_m_* (3)

where *Q_t_* is the adsorption capacity at equilibrium (mg/g); *C_t_* is the equilibrium concentration of the acetamiprid solution (mg/L); *t* (min) is the contact time; *X_m_* (mg/g) is the maximum monolayer adsorption capacity and *b* (L/mg) is the adsorption equilibrium constant.

The Langmuir isotherm model assumes that monolayer adsorption occurs on homogeneous surfaces containing a finite number of adsorption sites and no lateral interactions between the adsorbed molecules exist [38]. The dimensionless constant (*R_L_*) is a very important parameter from Langmuir isotherm which suggests that the removal of acetamiprid onto TPAC is favorable or not and can be determined by the following equation:

*R_L_* = 1/(1 + *bC*^0^) (4)

where *C*^0^ is the initial acetamiprid level (mg/L) and *b* is the Langmuir isotherm constant. *RL* value describes the adsorption mechanisms, which is unfavorable (*R_L_* > 1), linear (*R_L_* = 1), favorable (0 < *R_L_* > 1) or irreversible (*R_L_* = 0). The value of *R_L_* in the present investigation was found to be 0.09 indicating that the adsorption of acetamiprid onto TPAC is favorable.

**Figure 8:**
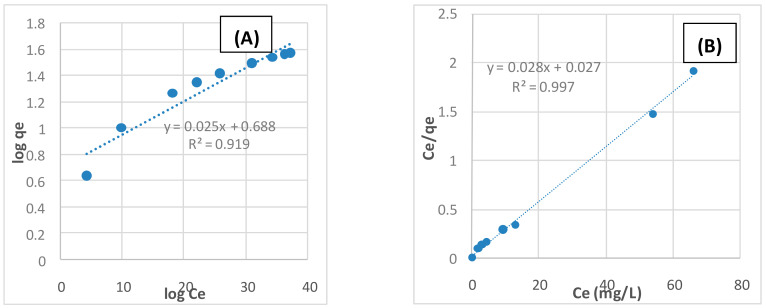
Adsorption isotherm models (**A**) Langmuir and (**B**) Freundlich.

The Freundlich isotherm model assumes that multilayer adsorption occurs on heterogeneous surfaces of the adsorbent [39]. In addition, assuming the stronger binding sites were occupied first, and the strength of the binding decreased with the increasing of the occupation of sites. The linear form of Freundlich isotherm is given by:

(5) $$\log\;q_{e}=\log\;K_{f}+1/n\;\log\;C_{e}$$

where *K_f_* is an indicator of the adsorption capacity of TPAC (i.e., the greater the value of *K_f_* the greater adsorption capacity). The value of 1/*n* is the heterogeneity factor of site energies and reflected the intensities of adsorption [40]. These suggested that the ability for adsorption of acetamiprid pesticide by TPAC was strong. In this study, different isotherms have been used to explain the adsorption mechanism. The isotherm parameters are presented in Table 3. The different isotherm plots for the adsorption of acetamiprid onto TPAC are shown in Figure 8A,B. The best fit of these isotherm models was selected according to the value of the correlation coefficients (*R*^2^). It was found that the Langmuir isotherm model is the best fit for the adsorption of acetamiprid, which indicated that the acetamiprid pesticide is adsorbed by TPAC as a monolayer with the homogeneous surface. The maximum adsorption capacity was found to be 35.71 mg/g.

The energy of adsorption was less than 8 KJ/mol indicating that the adsorption of acetamiprid onto TPAC is physical adsorption (Van der Waals forces). Similar trends have been reported by [18], they studied the adsorption of oxamyl pesticide onto Egyptian apricot stones.

### 2.5. Thermodynamics

Thermodynamic is essential to find out whether the process is spontaneous or not. The thermodynamic parameters such as enthalpy changes (*ΔH°*), entropy changes (*ΔS°*), (*K_D_*) binding affinity and Gibbs free energy changes (*ΔG°*) were used to evaluate the influence of temperature on adsorption process and determine the nature and spontaneity of the adsorption of acetamiprid pesticide onto TPAC were calculated by the following equations:

*ΔG° = −RT ln K_D_* (6)

*ln K_D_ = −ΔH°/RT + ΔG°/R* (7)

The values of thermodynamic parameters are in Table 4. The negative value of *ΔH°* confirmed the exothermic nature of the adsorption process. The value of *ΔH°* was found to be in the range between 8 and 40 KJ/mol which suggests the physico-sorption process [41]. The negative values of *ΔG°* were −71.7, −72.8 and −74.5 KJ/mol, respectively at all temperatures and indicate that the adsorption of acetamiprid by TPAC is spontaneous.

### 2.6. Comparison of Acetamiprid Adsorption Capacity with Other Adsorbents

The adsorption capacity of acetamiprid pesticide on TPAC was compared with different adsorbents reported in the previous studies. According to Table 5, the adsorption capacity in the present work of acetamiprid pesticide on tangerine peels-derived activated carbon was higher in comparison with some studies [13] while lower in comparison with other studies [23]. The reason for this variation is due to the adsorbent dose and the initial concentration used. The adsorption capacities varied from different adsorbents according to different parameters such as, source of the activated carbon, surface characterization, pH, particle size and functional groups.

### 2.7. Reusability of the Activated Carbon

**Figure 9:**
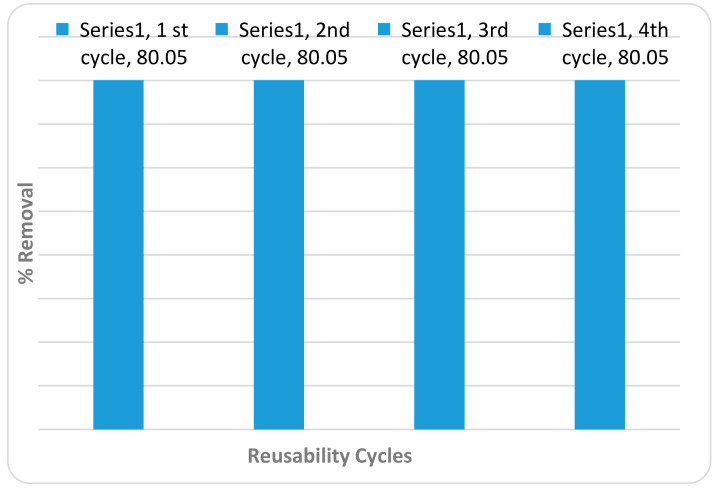
Reusability of TPAC.

Reusability or recycling is very important benefits of the adsorbent for the removal of different contaminants due to the lower cost. Figure 9. shows the performance of TPAC after four cycles of adsorption–desorption. The percentage removal of acetamiprid by TPAC was the same during each cycle. We measured 80.05% during the first cycle, 80.05% during the second cycle; 80.05% during the third cycle and 80.05% during the fourth cycle. These results indicated that the TPAC has been reused for four cycles at a commercial scale level in order to lower the cost of the adsorbing materials and reduce the need for new adsorbents.

## 3. Materials and Methods

### 3.1. Apparatus

The concentrations of acetamiprid in solution before and after the adsorption process was quantized using a reversed-phase high performance liquid chromatography (HPLC) (Agilent 1200, WA, USA) coupled with a variable wavelength diode array detector (DAD), C18 column (5 µm particles, 250 mm × 4.6 mm i.d.) and an auto-sampler with an electric sample valve. The mobile-phase solvent was 70% Milli-Q ultrapure water and 30% acetonitrile at a constant flow rate of 1.0 mL/min. The wavelength of measuring acetamiprid was 246 nm. The surface functional groups of TPAC before and after adsorption of acetamiprid pesticide were determined by Fourier Transform infrared spectrometer (PerkinElmer 1720). The textural characteristics of TPAC including surface area, pore volume and pore diameter were determined by BELSORP-mini II instrument. The X-ray diffraction of the prepared activated carbon was performed with a XPERT PRO (Almelo, The Netherlands) diffractometer in the range of 2θ (4–80°). Zeta plus Zeta potential analyzer (Nanozs, Malvern Company, Malvern, UK) determined the particle size of the prepared activated carbon. Prior to the analysis, all samples were degassed at around 200 °C under vacuum overnight to clean their surface.

### 3.2. Chemicals 

All chemicals used in this work were of analytical reagent grade. Acetamiprid (97% purity), acetonitrile (HPLC grade) and orthophosphoric acid (85%) were obtained from Sigma-Aldrich (St. Lois, MO, USA). Deionized water was obtained from a Millipore Milli-Q Academic Ultra-Pure Water Purification System.

### 3.3. Preparation of Activated Carbon

All tangerine peel samples were collected from the local fruit markets in Egypt. The peels were washed with distilled water and dried in an oven at 80 °C for 48 h. Chemical activation was carried out by impregnation of the tangerine peels with 1:1-(*w*/*w*) H_3_PO_4_ for 24 h then dried at 100 °C for further 24 h. Pyrolysis was carried out in a muffle furnace at 500 °C in the absence of air for 2 h. The produced activated carbon after pyrolysis was washed with deionized water and dried in an oven at 80 °C for 24 h. The yield of the produced activated carbon was 48.4% and calculated by:

*Yield*, % = (*Dry weight of the activated carbon/Dry weight of the precursor*) × 100 (8)

### 3.4. Acetamiprid Uptake Study

A 100 mg of TPAC adsorbent was inserted into 100-mL aliquots of acetamiprid solutions (10–100 µg/mL). The solutions were stirred for 240 min at room temperature and filtered. Acetamiprid concentrations, before and after the treatment, were measured using HPLC measurements. The removal percentage of acetamiprid was calculated using the following equation:

*Removal*% = [(*C*_0_ − *C_t_*)/*C*_0_] × 100 (9)

where: *C_0_* and *C_t_* are the acetamiprid concentration in µg/mL at initial and after time *t*, respectively.

### 3.5. Batch Adsorption Experiments

Aliquots (100 mL) of 25-µg/mL acetamiprid solution were treated with different adsorbent doses (0.02–0.2 gm), under different pH values (2–8) for varying contact times (10–300 min). The acetamiprid solutions were stirred, filtered and the concentration was measured using HPLC measurements.

### 3.6. Adsorption Isotherms and Kinetics

Adsorption equilibrium was performed using the different initial concentrations of acetamiprid (10–100 µg/mL) in presence of 0.1 g of TPAC under constant stirring at 200 rpm. The equilibrium was analyzed using different isotherm models such as Langmuir, Freundlich and Dubinin–Radushkevich (D-R) adsorption isotherm models. The adsorption capacity of acetamiprid by TPAC was calculated from the following equation:

(10) $$q_{e}=\frac{\left(C_{0}-C_{e}\right)\;V}{m}$$

The adsorption kinetics data were evaluated with 0.1 g of TPAC at initial concentration of acetamiprid of 25 µg/mL and the samples were taken after time varied from 10 to 300 min.

### 3.7. Adsorption Thermodynamics

Adsorption of acetamiprid was investigated at different temperatures in the range 25–50 °C using initial concentration 25 µg/mL and 0.1 g of TPAC. Prior to the analysis, the samples were centrifuged at 4000 rpm for 5 minutes and filtered using 0.45-µm Millipore filter. Then the concentration of acetamiprid was determined by HPLC method.

### 3.8. Reusability

The reusability TPAC was tested by performing five alternating adsorption–desorption cycles. Each adsorption cycle was performed as mentioned previously then the adsorbent was filtered, washed and dried. The collected adsorbent was then dispersed into 20 mL of 0.5-M HCl and shaken at 180 rpm for 10 minutes to regenerate the adsorbent. Prior to the next cycle, the regenerated adsorbent was washed repeatedly with ultrapure water until the effluent was neutral (pH 7). The regenerated TPAC was used for sequential adsorption–desorption cycles and the acetamiprid adsorption efficiency was obtained.

## 4. Conclusions

Tangerine peels-derived activated carbon (TPAC) was synthesized and characterized by FTIR, surface area, XRD and particle size analysis. TPAC was applied to the removal of acetamiprid pesticide from aqueous solution. The adsorption of acetamiprid was influenced by adsorbent dose, pH, initial concentration, contact time and temperature. The adsorption isotherm was well fitted by Langmuir with a maximum adsorption capacity of 35.71 mg/g. The adsorption process was found to be rapid in the first 20 min and attained equilibrium at 240 min. The pseudo-second-order kinetic model describes the adsorption of acetamiprid by TPAC better than the pseudo-first-order kinetic model. The thermodynamics indicates that the adsorption of acetamiprid onto TPAC was exothermic and spontaneous in nature. Furthermore, the potential of regenerating of the tangerine peels activated carbon was investigated and successfully regenerated and reused for three cycles. It is concluded that the tangerine peels activated carbon could be a good activated carbon for the removal of acetamiprid pesticide from aqueous media.

## Figures and Tables

**Table 1:** Textural features and elemental analysis of TPAC before (A) and after (B) acetamiprid adsorption.

Samples	Mesopore Size, nm	Micropore Size, nm	Mesopore Volume, cc/g	Micropore Volume, cc/g	Total Pore Volume, cc/g	Mesopore Area, m^2^/g	Micropore Area, m^2^/g	Total Surface Area, m^2^/g
TPAC (A)	3.6	1.61	0.47	0.17	0.64	385.9	301.9	687.8
**TPAC (B)**	3.7	1.6	0.35	0.03	0.38	196.4	99.9	296.4

**Table 2:** Kinetic parameters and correlation coefficients for the removal of acetamiprid by TPAC.

Kinetic Model	Parameter	Values
Pseudo-first-order	*k*_1_(1/min)	0.01 ± 0.02
	*q_e_* (mg/g)	7.3 ± 0.01
	*R* ^2^	0.884
Pseudo-second-order	*k_2_* (g/mg.min)	0.005 ± 0.001
	*q_e_* (mg/g)	23.3 ± 0.717
	*R* ^2^	0.998

**Table 3:** Parameters of isotherm models for the removal of acetamiprid by TPAC.

Isotherm Model	Parameter	Values
Freundlich	*K_f_*	4.8 ± 0.03
	*R* ^2^	0.919
	1/*n*	0.02 ± 0.001
Langmuir	*B* (L/mg)	1.01 ± 0.02
	*q_m_* (mg/g)	35.7 ± 0.05
	*R* ^2^	0.997

**Table 4:** Thermodynamic parameters for the adsorption of acetamiprid pesticide onto TPAC.

Temperature (K)	Δ*G*° (KJ/mol)	Δ*H*° (KJ/mol)	Δ*S*° (KJmol^−1^ K^−1^)
298	−71.7	−37.8	−113.5
308	−72.8		
323	−74.5		

**Table 5:** Comparison of acetamiprid removal by different adsorbents.

Adsorbent Type	Maximum Adsorption Capacity (mg/g)	Initial Concentration (mg/L)	Contact Time (min)	Optimum pH	Isotherm Fitted	Adsorbent Dose (g)	% Removal	Ref.
Bentonite	9.1	100	30	7.0	Langmuir	1	–	[13]
Bentonite and clay	7.8	100	30	7.0	Langmuir	1	–	[13]
Kaolin	7.7	100	30	7.0	Langmuir	1	–	[13]
Orange peels activated carbon	151.5	300	120	5.6	Freundlich	1	99.4	[23]
Almond shells activated carbon	370.3	300	120	5.6	Freundlich	0.5	99.4	[23]
Tangerine peels activated carbon	35.7	25	240	5.6	Langmuir	0.1	92.0	This Study
